# Nitric Oxide Is a Mediator of Antiproliferative Effects Induced by Proinflammatory Cytokines on Pancreatic Beta Cells

**DOI:** 10.1155/2013/905175

**Published:** 2013-06-12

**Authors:** Laura Quintana-Lopez, Manuel Blandino-Rosano, Gonzalo Perez-Arana, Alberto Cebada-Aleu, Alfonso Lechuga-Sancho, Manuel Aguilar-Diosdado, Carmen Segundo

**Affiliations:** ^1^Investigation Unit, Puerta del Mar Hospital, Cadiz, Spain; ^2^Pediatric Endocrinology Unit of Paediatric Service, Puerta del Mar Hospital, Cadiz, Spain; ^3^Endocrinology and Nutrition Service, Puerta del Mar Hospital, 11009 Cadiz, Spain; ^4^“Salus Infirmorum” Faculty of Nursing, Cadiz University, Spain

## Abstract

Nitric oxide (NO) is involved in several biological processes. In type 1 diabetes mellitus (T1DM), proinflammatory cytokines activate an inducible isoform of NOS (iNOS) in **β** cells, thus increasing NO levels and inducing apoptosis. The aim of the current study is to determine the role of NO (1) in the antiproliferative effect of proinflammatory cytokines IL-1**β**, IFN-**γ**, and TNF-**α** on cultured islet **β** cells and (2) during the insulitis stage prior to diabetes onset using the Biobreeding (BB) rat strain as T1DM model. Our results indicate that NO donors exert an antiproliferative effect on **β** cell obtained from cultured pancreatic islets, similar to that induced by proinflammatory cytokines. This cytokine-induced antiproliferative effect can be reversed by L-NMMA, a general NOS inhibitor, and is independent of guanylate cyclase pathway. Assays using NOS isoform specific inhibitors suggest that the NO implicated in the antiproliferative effect of proinflammatory cytokines is produced by inducible NOS, although not in an exclusive way. In BB rats, early treatment with L-NMMA improves the initial stage of insulitis. We conclude that NO is an important mediator of antiproliferative effect induced by proinflammatory cytokines on cultured **β** cell and is implicated in **β**-cell proliferation impairment observed early from initial stage of insulitis.

## 1. Introduction

Type 1 diabetes mellitus (T1DM) is characterized by a loss of beta cell mass due to an autoimmune process. In a phase prior to the onset, immune cells infiltrate the islets creating an inflammatory microenvironment responsible for the *β*-cell-specific toxicity. Proinflammatory cytokines, such as interleukin-1 beta (IL-1*β*), tumor necrosis factor alpha (TNF-*α*), and interferon gamma (IFN-*γ*), secreted by activated lymphocytes and macrophages during insulitis, induce NFkB mediated iNOS expression which has a key role in *β*-cell apoptosis in the early T1DM stage [1].

Nitric oxide is a ubiquitous molecule which acts as messenger in diverse biological processes. It is produced in tissues by the enzyme nitric oxide synthase (NOS) by oxidation of the amino acid L-arginine. There are three different tissue-specific NOS isoforms: Ca^2+^/calmodulin-independent and inducible NOS (iNOS), neuronal NOS (nNOS), and endothelial NOS (eNOS). These last two isoforms also exhibit constitutive expression and Ca^2+^/calmodulin-dependent activity. 

Expression of NOS isoforms has been studied in pancreatic islets, and the nNOS form has been observed in *β*-cell cells, located, mainly, in insulin secretory granules. It exhibits cytochrome C reductase activity in addition to NO production activity, and a balance between both functions is essential for a normal insulin secretion in response to glucose stimulation. In contrast, eNOS expression has not been found in rat islets under basal conditions [2, 3]. 

Several mechanisms have been proposed in explaining NO-mediated *β*-cell cell apoptosis. Prooxidant agents formed from NO induce damages in DNA which activate p53 which induces proapoptotic expression of genes such as BAX, FAS, NOXA, and PUMA. A p53-independent mechanism has been described in which NO acts by inhibiting the sarcoendoplasmic reticulum pump Ca^2+^ ATPase 2b (SERCA2b) protein and, subsequently, by inducing the endoplasmic reticulum stress mechanisms [4, 5]. The importance of the mitochondrial pathway has also been highlighted in NO-mediated *β*-cell cell apoptosis [6, 7]. Paradoxically, an antiapoptotic effect of low concentration of NO has been reported in several systems, including *β* cells [8–12]. 

In addition to apoptosis, cell proliferation has been described as a NO-regulated process. Although certain activating effects have been reported in physiological systems [13, 14], the main role of NO in cellular proliferation is inhibitory. In the subventricular zone, NO induces inhibition of stem cell proliferation by a nitrosylation process [15]. Other proposed mechanism for NO antiproliferative action is a G1-S inhibition mediated by an induction of the cell cycle inhibitor p21 [16] or cyclins inhibition [17]. Very few studies have examined the role of NO in proliferation in *β* cells. Recently, NO-mediated neogenesis stimulation has been observed in an alloxan-induced murine model of diabetes [18].

A proinflammatory cytokine-mediated inhibition of cultured *β*-cell proliferation in addition to the apoptotic effect has been observed by our group. As described previously, NO production is one of the most important events mediated by proinflammatory cytokines. Therefore, the aim of this study was to assess the importance of NO in the cytokine-mediated antiproliferative effect on cultured *β* cells and to determine the role(s) of different NO synthase isoforms present in pancreatic islets.

## 2. Methods

### 2.1. Animals

All animal procedures were performed with the approval of the Cádiz University School of Medicine (Cádiz, Spain) Committee for the Ethical Use and Care of Experimental Animals.

Bio-Breeding (BB) and Wistar rats were kept under conventional conditions in an environment-controlled room (20-21°C, 12 h light-dark cycle) with water and standard laboratory rat chow available *ad libitum* and their weight was daily recovered. Blood extracted from the tail vein was used in BB rats for weekly random glucose measurements using an automatic glucose monitor (Accu-Chek Optimum, Roche Diagnostic, Basel, Switzerland).

### 2.2. Isolation and Culture of Rat Islets

Pancreatic islets were isolated from adult male Wistar rats as described previously by McDaniel et al. [19]. Isolated islets were cultured in RPMI medium (Sigma-Aldrich, St. Louis, MO, USA) supplemented with 2 mM L-glutamine (Gibco Invitrogen, Carlsbad, CA, USA), 100 U/mL penicillin, 100 *μ*g/mL streptomycin (Pen-Strep, Bio-Whittaker Europe, Verviers, Belgium), and 10% fetal bovine serum (FBS, Gibco Invitrogen, Carlsbad, CA, USA). Glucose concentration used was 5.5 mM. Proinflammatory cytokines (PeproTech EC Ltd, London, UK) used in the experiments were recombinant human IL-1*β* (50 U/mL), recombinant rat IFN-*γ* (1000 U/mL), and recombinant rat TNF-*α* (1000 U/mL). These concentrations were selected as being appropriate based on the results of previous published studies [20, 21].

### 2.3. Culture Treatment

Pancreatic islet cultures were treated with different drugs related to NO metabolism. NO donors, S-nitroso-N-acetyl-DL-penicillamine (SNAP), and diethylenetriamine/nitric oxide adduct (DETA-NO), obtained from Sigma-Aldrich (St. Louis, MO, USA) show different NO release rates. DETA-NO is a member of the NONOates family and has a half-life (*t*_1/2_) of 20 h at 37°C, pH 7.4. It generates 2 moles of NO following simple first-order kinetics. The *t*_1/2_ of SNAP is approximately 6 h at 37°C and pH 6–8 although its biological activity is highly influenced by the molecular environment of the parent thiol. Inhibitors of NOS 7-nitroindazole (7-Ni), N-[[3-(aminomethyl)phenyl]methyl]-ethanimidamide, dihydrochloride (1400 W), and N5-(1-iminoethyl)-L-ornithine, dihydrochloride (L-NIO), purchased from Sigma-Aldrich (St. Louis, MO, USA) were used to inhibit the activity of neuronal, inducible, and endothelial isoforms of NOS, respectively.

### 2.4. Proliferation Assays in Cultured Islets

Proliferating *β* cells were detected using 5-bromo-2′-deoxyuridine (BrdU) 5 *μ*mol/L label (Sigma-Aldrich, St. Louis, MO, USA) which was added to the cultures from the start of the assay together with the drugs being tested, when appropriate. Following the appropriate culture duration, the islets were recovered and incubated for 15 min with trypsin-EDTA (0.25% trypsin, 1 mM EDTA) in Hanks' balanced salt solution without Ca^2+^ and Mg^2+^ (Gibco Invitrogen, Carlsbad, CA, USA) at 37°C, and the islets were gently dispersed. After washing with PBS, cells were cytospin on poly-L-lysine-coated slides and fixed in 4% methanol-free formaldehyde. Slides were immuno-stained using monoclonal mouse anti-BrdU (Dako Cytomation, Denmark) and polyclonal guinea pig anti-insulin (Sigma-Aldrich, St. Louis, MO, USA) antibodies, according to the manufacturer's instructions. Cells were permeabilized by incubation for 30 min with 0.1% Triton-X100 in PBS and washed twice with 100 mM glycine buffer containing 0.1% Triton-X100 and 3% bovine serum albumin. Cells were then treated with HCl (2N) in PBS for 30 min, neutralized with borax/borate buffer (0.1 M, pH 8.9) for 30 min, washed, and incubated overnight at 4°C with anti-BrdU and anti-insulin antibodies. Stained cells were revealed using anti-mouse IgG antibody (alexa-546 conjugated) and anti-guinea pig IgG (alexa-488 conjugated) antibody (Molecular Probes Inc, Eugene, OR, USA). Cell nuclei were stained with 4′-6-diamidino-2-phenylindole (DAPI). To determine the proliferating fraction, total and insulin-positive/BrdU-positive cells were analyzed using a fluorescence microscope in randomized conditions, by a single investigator. For statistical purposes, the percentage of positive cells was calculated for each incubation condition.

### 2.5. Immunofluorescence Staining of Cultured Pancreatic Islets

48 h cultured islets in presence and absence of cytokines were processed for histology study by histogel and paraffin inclusion. 10 *μ*m sections were stained using a polyclonal rabbit anti-iNOS (ab15323, Abcam, Cambridge, UK) and a polyclonal guinea pig anti-insulin (Sigma-Aldrich, St. Louis, MO) and examined using a microscope equipped with a digital camera and the image analysis CellDsoftware (Olympus, Hamburg, Germany).

### 2.6. Treatment Protocol

Randomly grouped BB rats daily received an intraperitoneal injection of vehicle (0.1% DMSO diluted in injection water) alone or containing L-NMMA at a dose of 30 mg/kg, from 4 to 9 weeks of age. Animals were sacrificed at 4 (before treatment), 7, and 9 weeks of age.

### 2.7. IPGTT Assay

Wistar, BB, and thymectomized BB rats were fasted overnight (16–18 h) and a blood sample was collected from the tail vein (fasting or 0 min sample). Then, an intraperitoneal injection of 40% solution of glucose was administered (2 g/kg), followed by blood sampling at 15, 30, 60, and 120 min after the glucose administration. Glycemia was measured with an automatic glucose monitor (Accu-Chek Optimum, Roche Diagnostic, Basel, Switzerland). Blood samples obtained from fasting and 15 min after glucose administration were also used to plasma extraction and insulin quantification by ELISA technique (Insulin rat ultrasensitive kit. ALPCO, Salem, MA, USA).

### 2.8. Quantification of *β*-Cell Mass

7 and 9 weeks of age BB rat pancreas were resected, weighed, fixed in Bouin's solution for 6 h, and postfixed in formalin for 12 h. Then, they were dehydrated, paraffin embedded, and longitudinal 10 *μ*m microtome sections were obtained. Insulin was stained in obtained pancreas by immunohistochemical techniques using a mouse anti-rat insulin monoclonal antibody and a peroxidase conjugated goat anti-mouse IgG antibody and revealed with DAB kit (Sigma Aldrich, St. Louis, MO, USA). Insulin-positive areas were quantified in two complete sections of each pancreas using a microscope equipped with a digital camera and the image analysis Cell D software (Olympus, Hamburg, Germany). The investigators were blinded with respect to the provenance of the samples. *β*-cell mass values were calculated by multiplying the total insulin-positive area/total pancreatic area ratio by the total pancreas weight.

### 2.9. Western Blotting

Equivalent numbers of islets under the different experimental conditions described previously were lysed in lysis buffer (125 mM Tris-HCl buffer pH 6–8, 2% SDS, 1 mM DTT and containing protease and phosphatase inhibitors). Protein (40 *μ*g) was loaded and electrophoresed on 8% SDS-PAGE. Proteins were transfer-blotted on to polyvinylidene fluoride (PDFV) membranes and then incubated in blocking buffer (5% nonfat milk in 10 mM Tris-HCl, 1.15 M NaCl and 0.1% Tween-20) for 1 h at room temperature. The blots were then incubated with rabbit polyclonal antibody against eNOS (Santa Cruz Biotechnology, Santa Cruz CA, USA) and iNOS (Transduction Laboratories BD, NJ, USA) overnight according to the manufacturer's instructions, followed by incubation with peroxidase conjugated anti-rabbit IgG for 1 h at room temperature. Stained bands were revealed using Immun-Star Western C kit (Bio-Rad, Hercules CA, USA) and quantified by Quantity One software Version Upgrade (Bio-Rad, Hercules, CA, USA). *β*-tubulin expression was used as loading control and eNOS expression was calculated as ratio of eNOS: *β*-tubulin densities.

### 2.10. Histological Examination of *β*-Cell Infiltration

Histological examination of 7 and 9 weeks of age rat pancreatic islets was performed in Harris' H&E stained pancreas sections using ×20 objective lens. The severity of insulitis was graded as a function of the mononuclear cell infiltration of the pancreatic islets: 0 = no infiltrate; 1 = periductular infiltrate; 2 = periislet infiltrate; 3 = intraislet infiltrate; 4 = intraislet infiltrate associated with *β*-beta cell destruction. Thirty islets were examined in each pancreas and the mean score was calculated by dividing the total score by the number of islets examined. 

### 2.11. Statistical Analysis

Results are presented as means ± SEM of measurements performed in at least 3 animals. Statistical comparisons were performed by Mann-Whitney test. All *P* values ≤ 0.05 were considered statistically significant.

## 3. Results

### 3.1. Effect of NO Donors on *β*-Cell Proliferation in Cultured Pancreatic Islets

**Figure 1:**
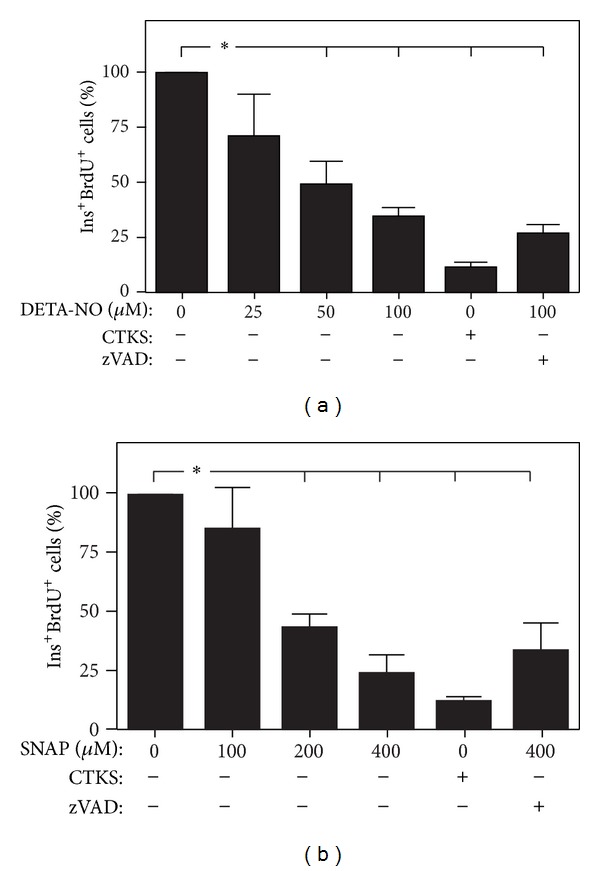
Effect of NO donors in cultured beta cell proliferation. Rat islets were cultured for 48 h and treated with NO donors DETA-NO (a) and SNAP (b) at increasing concentrations, alone or in combination with zVADfmk (100 *μ*M), a caspase inhibitor, and with the combination of proinflammatory cytokines IL-1*β* (50 U/mL) + IFN-*γ* (1000 U/mL) + TNF-*α* (1000 U/mL) (CTKS). Cultures were stained with BrdU over the course of the culture period. Bar graphs are percentage means ± SEM of accumulated BrdU/insulin-positive cells over the culture period relative to insulin-positive cells. Values are the means of 4 experiments. *P < 0.05 treated versus control.

To determine the role of NO donors on *β*-cell proliferation, pancreatic islets were treated with proinflammatory cytokines or different concentrations of NO donors SNAP and DETA, alone or in combination with the caspase-3 inhibitor z-VAD-fmk. *β*-cell proliferation measured by BrdU incorporation showed that the NO donors, DETA-NO (Figure 1(a)) and SNAP (Figure 1(b)) exert an antiproliferative action on *β* cells in a dose-dependent manner. This antiproliferative effect was similar to that obtained by proinflammatory cytokines. This effect of NO donors was not modified by addition of z-VAD-fmk to the cultures.

### 3.2. Role of NO in Antiproliferative Effect of Proinflammatory Cytokines on Pancreatic *β* Cells

**Figure 2:**
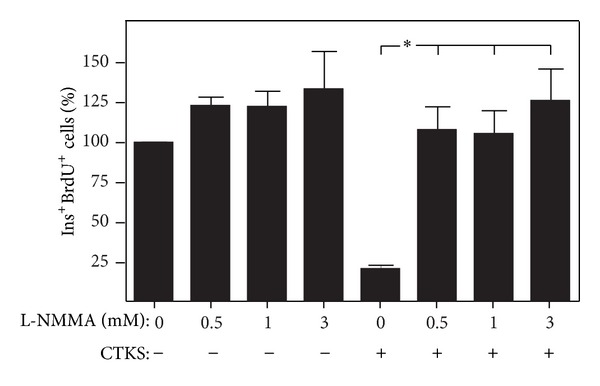
Effect of NO inhibition on islet *β*-cell proliferation. Rat islets were cultured for 48 h in the presence of cytokines IL-1*β* (50 U/mL) + IFN-*γ* (1000 U/mL) + TNF-*α* (1000 U/mL) (CTKS) alone or in combination with NOS inhibitor L-NMMA and stained with BrdU over the course of the culture period. Results are presented as percentage means ± SEM of combined BrdU/insulin positive cells relative to insulin-positive cells in a minimum of 5 experiments. *P < 0.05 treated versus control.

To determine the contribution of NO to the antiproliferative effect of proinflammatory cytokines on pancreatic *β* cells, pancreatic islets were cultured over a 48 h period and treated with proinflammatory cytokines, alone or in the presence of L-NMMA (an inhibitor of nitric oxide synthase). Inhibition of *β*-cell proliferation induced by proinflammatory cytokines was completely abolished by L-NMMA treatment (Figure 2).

### 3.3. Role of Guanylate Cyclase in the NO Effect on *β*-Cell Proliferation

**Figure 3:**
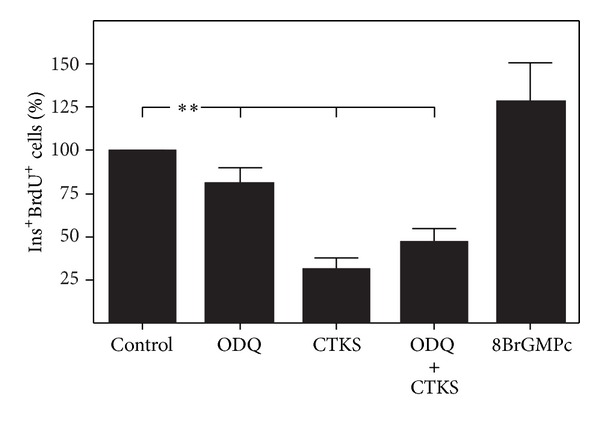
Effect of cGMP pathway alterations in *β*-cell proliferation in response to proinflammatory cytokines. Rat islets were cultured for 48 h in the presence of cGMP pathway activator 8BrGMPc (100 *μ*M) and a mixture of cytokines IL-1*β* (50 U/mL) + IFN-*γ* (1000 U/mL) + TNF-*α* (1000 U/mL) (CTKS) alone or in combination with ODQ (10 *μ*M), an inhibitor of guanylate cyclase. Results are presented as percentage means ± SEM of combined BrdU/insulin-positive cells relative to insulin positive cells in a minimum of 5 experiments. **P < 0.01 treated versus control.

Guanylate cyclase is the enzyme through which NO exerts the greater part of its effect. To assess the implication of the guanylate cyclase pathway in the NO effect on *β*-cell proliferation, cultures of pancreatic islet were treated for 48 h with 8Br-cGMP (a guanylate cyclase analogue) and its inhibitor ODQ, alone or in combination with proinflammatory cytokines. As shown in Figure 3, the cGMP analogue exerts no effect on *β*-cell proliferation, and the antiproliferative effect of cytokines is not altered by the cGMP inhibitor ODQ. 

### 3.4. Involvement of Different Isoforms of NOS in Proinflammatory Cytokine-Induced Decrease on *β*-Cell Proliferation

**Figure 4:**
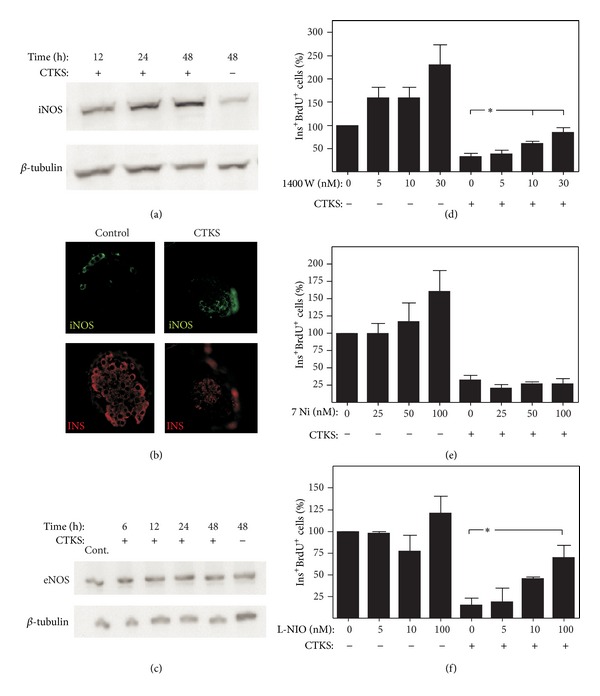
Role of different NOS isoforms in cytokine-induced antiproliferation in cultured *β* cells. (a) and (c) iNOS and eNOS expression was assessed using western blot analysis in pancreatic islets cultured over 48 h under basal conditions and treated with a mixture of cytokines IL-1*β* (50 U/mL) + IFN-*γ* (1000 U/mL) + TNF-*α* (1000 U/mL) (CTKS) over 6, 12, 24, and 48 h. A representative image is shown (Cont: positive brain control in eNOS Western blot). (b) Representative image of iNOS (green) and insulin (red) immunostaining of control and cytokines treated (CTKS) pancreatic islets cultured over 48 h. (d), (e), and (f) rat islets cultured over 48 h in the presence of 7 Ni (e), 1400 W (d), and L-NIO (f) alone or with the mixture of cytokines (CTKS). Results are presented as percentage means ± SEM of combined BrdU/insulin-positive cells relative to insulin-positive cells in a minimum of 5 experiments. *P < 0.05 treated versus control.

To study the role of constitutive and inducible NOS isoforms in the antiproliferative action of cytokines, cultured pancreatic islets were treated with proinflammatory cytokines in addition to 1400 W and 7-Ni, inhibitors of inducible and constitutive isoforms of nitric oxide synthase, respectively; the resultant *β*-cell proliferation was quantified. The antiproliferative effect of proinflammatory cytokines underwent no modification when islet cultures were incubated with the constitutive NOS inhibitor (Figure 4(e)). Related to iNOS, cytokine-dependent induction of protein was observed from 24 h of culture (Figure 4(a)). iNOS expression study by immunohistochemical techniques in control cultured pancreatic islets showed a scarce stain with peripheral profile. However, cultured islet in presence of cytokines showed an increased iNOS expression localized internally in the islet (Figure 4(b)). The specific inhibitor 1400 W partially reverted cytokine-induced decreased proliferation in a dose-dependent manner (Figure 4(d)). Endothelial isoform of NOS expression was initially examined in pancreatic islets cultured under control condition, or after stimulation with proinflammatory cytokines. As shown in Figure 4(c), eNOS isoform was expressed in pancreatic islets and was not altered by exposure to proinflammatory cytokines. To study the role of eNOS on the antiproliferative effect of cytokines, L-NIO (an inhibitor with higher affinity for eNOS than the other isoforms) was added to cultures in addition to cytokines, and *β*-cell proliferation was quantified. A partial inhibition of the antiproliferative effect of proinflammatory cytokines was observed in treated cultures (Figure 4(f)) mediated by L-NIO.

### 3.5. Effect of Early Treatment with L-NMMA on BB Rats Weight and Glucose Homeostasis during Insulitis Stage

**Figure 5:**
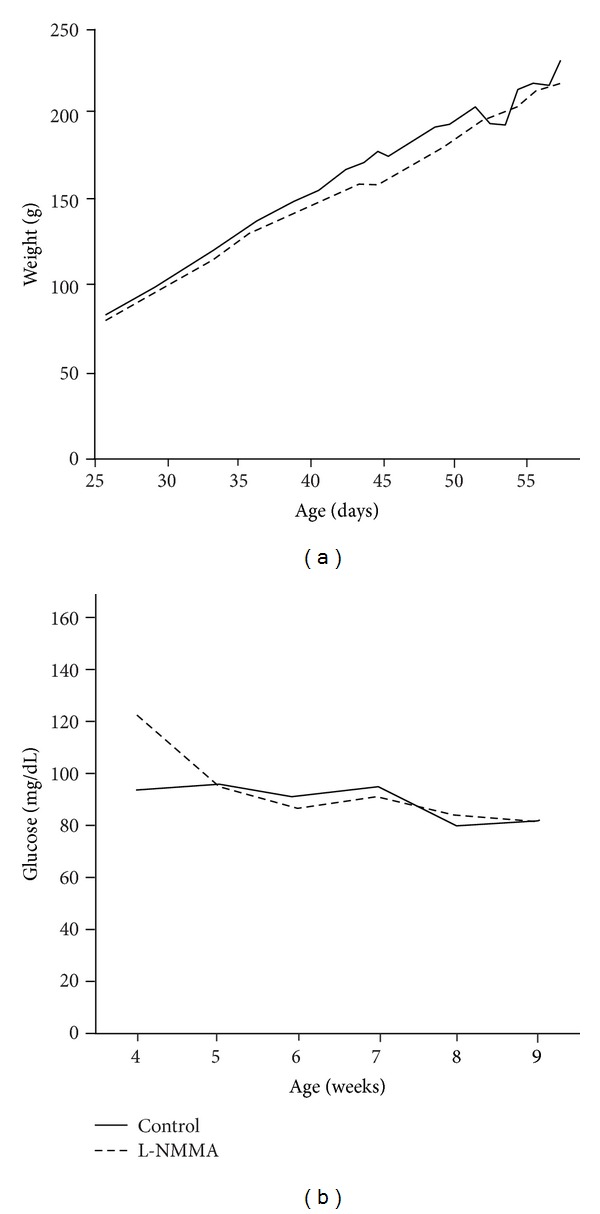
(a) Body weights were daily recorded in untreated (solid line) and L-NMMA-treated (dotted line) BB rats between 4 and 9 weeks of age. (b) Random blood glucose levels were weekly determined in untreated (solid line) and L-NMMA-treated (dotted line) BB rats between 4 and 9 weeks of age. The results are presented as means of weight (g) and glycemia (mg/mL) in a *n* = 5 animals.

**Figure 6:**
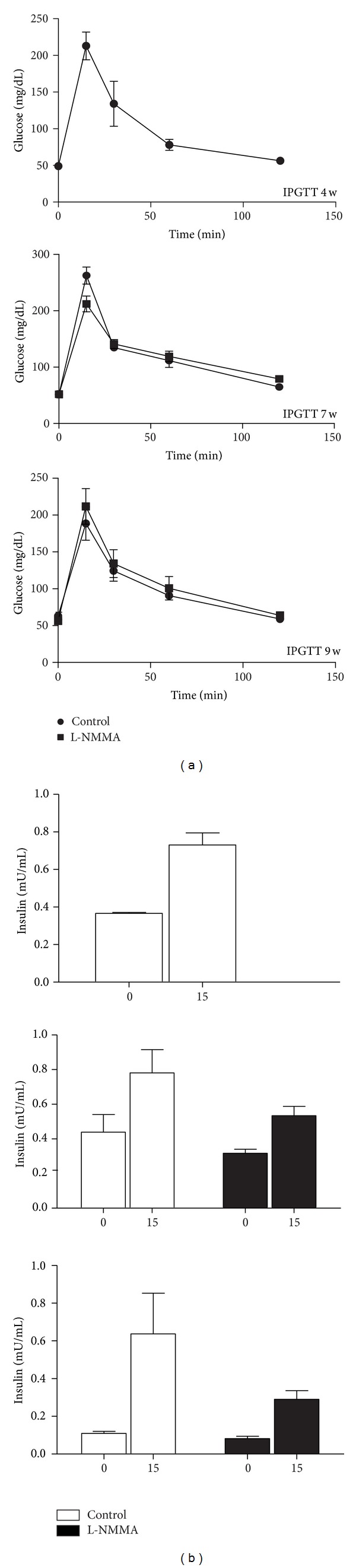
(a) IPGTT was performed in BB rats treated with vehicle (circle) or L-NMMA (square) at 4, 7, and 9 weeks of age. The results are presented as means ± SEM of glycemia (mg/mL) at the stated times after glucose injection in an *n* = 5 animals. (b) plasma insulin was quantified in blood samples obtained from BB rats treated with vehicle (white bars) or L-NMMA (black bars) at 4, 7, and 9 weeks of age. The results are presented as means ± SEM of insulin concentration (mU/mL) at fasting and 15 min after glucose injection in an *n* = 5 animals.

Weight and random glycemia monitoring along treatment period show no differences in those parameters between control and L-NMMA-treated animals. Glucose homeostasis was studied by IPGTT and insulin determination before and 15 min after intraperitoneal glucose administration. As shown in Figure 5, IPGTT displays a normal curve at studied times in L-NMMA treated as in control animals (Figure 6(a)). In addition, insulin quantification also shows no changes between control and treated animals at none of studied times (Figure 6(b)).

### 3.6. Effect of Early Treatment with L-NMMA on *β*-Cell Mass in BB Rats during Insulitis Stage

**Figure 7:**
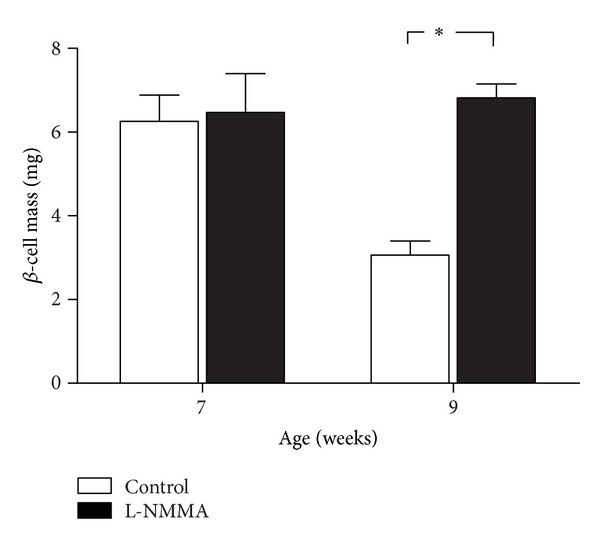
*β*-cell mass was determined in pancreatic sections from BB rats treated with vehicle (white bars) or L-NMMA (black bars) at 7 and 9 weeks of age. *β*-cell mass is presented in the bar graph as means ± SEM of values calculated as the ratio of insulin-positive area/total pancreatic area multiplied by the total pancreatic weight. Values are obtained from a mean of 5 animals. *P < 0.05 treated versus control.

BB rats treated with L-NMMA from 4 to 7 or 9 weeks of age were sacrificed and pancreas was removed from each rat to quantify beta cell mass by immunohistological techniques. In Figure 7, a beta cell mass loss undergone by BB rats between 7 and 9 weeks of age can be observed. L-NMMA reverts this effect at both studied times.

### 3.7. Effect of Early Treatment with L-NMMA on Infiltration Levels during Insulitis Stage

**Figure 8:**
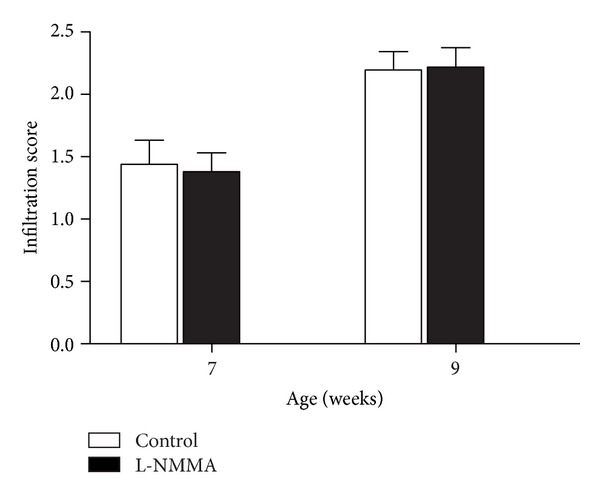
Infiltration scores were determined in Harris' H&E stained pancreatic sections of BB rats between 7 and 9 weeks of age treated with vehicle (white bars) and L-NMMA (black bars). Results are expressed as the means ± SEM of infiltration scores derived from a mean of 5 animals.

To test the effect of L-NMMA treatment on infiltration levels, H&E-stained sections obtained from treated and control BB rats were evaluated and infiltration scores were calculated. No changes were observed in infiltration level in response to L-NMMA at none of studied times (Figure 8).

## 4. Discussion

Currently, NO is considered an important mediator of cell signaling via its classical guanylate cyclase pathway or by exerting posttranslational modifications on an increasing number of proteins and hence regulating their activity. In the present study, we analyzed the role of NO in the previously described antiproliferative action of proinflammatory cytokines on pancreatic *β* cells. Different NO donors have been tested with respect to apoptosis induction in *β* cells. Loss of membrane integrity and cytochrome C release induced by DETA-NO and SNAP in insulin producing cell lines has been reported [22, 23]. In our system, NO donors SNAP and DETA-NO induced a decrease in cultured *β*-cell proliferation at concentrations up to 50 *μ*M for DETA-NO and up to 400 *μ*M for SNAP. This effect is dose dependent and similar to that obtained with proinflammatory cytokines (IL-1*β* + TNF-*α* + IFN-*γ*) treatment (Figure 1). The antiproliferative effect observed in response to NO donors is not dependent on the biological *t*_1/2_ and suggests that NO exerts its biological action in the first hours after exposure. To discard the possibility that the decrease in proliferation was due to a cytokine-induced apoptosis in proliferating *β* cell, we treated pancreatic *β*-cell cultures with the caspase-3 inhibitor zVADfmk, in addition to the NO donors. *β*-cell apoptosis inhibition induced by zVADfmk exerts no effect on the antiproliferative effect of NO donors suggesting that the two processes are unrelated.

The mechanism by which NO induces *β*-cell apoptosis has been extensively studied. Although studies in neonatal rat islets indicated that cytokine-induced NO production regulates the expression of up to 42 proteins, a considerable number of proteins are regulated in a NO-independent manner [24]. Thus, the cytokine-induced apoptosis in mouse *β* cells is not caused by the inducible isoform of NOS alone [25]. Endoplasmic reticulum stress has been described as a possible mechanism by which NO induces apoptosis [26]. Its presence regulates unfolded protein response (UPR) signaling pathway that determines apoptotic response to reticulum stress [27]. In addition to cytokines, there is evidence of NO participation in mitochondrial dysfunction and cell death occurring in PC12 cells in response to amyloid *β* [28]. 

Results from our study suggest that, in addition to apoptosis, NO can be implicated in the antiproliferative effect of proinflammatory cytokines on pancreatic *β* cell since treatment with NOS inhibitor L-NMMA is capable of reverting this cytokine-induced effect (Figure 2). Very few studies have investigated the possible mechanisms underlying the effect of NO on cellular proliferation. In pulmonary microvascular smooth muscle cells, exogenous NO upregulates p21, a protein which mediates cell cycle arrest [16]. Further, NO is capable of inhibiting ERK 1/2 and AKT activation in breast cancer cell lines and pancreatic *β* cells [29, 30]. These results are in accord with those observed by our group; that is, proinflammatory cytokines induced an inhibition of ERK 1/2 in islet cultures [31] and in early stages of insulitis [32]. However, NO has also been described as an inducer of proliferation, when acting at low concentrations. For example, in mesangial cells, the NO donor SNAP increases proliferation at concentrations of up to 200 *μ*M and inhibits proliferation at higher concentrations, in a process implying AKT signaling and cox-2 protein [33].

Activation of NO-sensitive guanylyl cyclase by NO binding to the enzyme's prosthetic heme group has been described as one of the most important NO-mediated cellular effects [34]. Nevertheless, results observed in our system suggest that this pathway is not involved in the NO-mediated antiproliferative effect observed in *β* cells from islets treated with proinflammatory cytokines (Figure 4). These results are in accord with those previously reported in tumor cell lines in which the proliferative effect of low NO concentration is mediated by the cGMP pathway, but the antiproliferative effect induced by high concentrations of NO is independent of this pathway [14]. A proposed cGMP-independent mechanism of NO action is its direct interaction with protein and, as such, performing posttranslational modifications such as nitration or s-nitrosylation. For example, s-nitrosylation of EGF receptor has been reported in human neuroblastoma NB69 as a mechanism of NO-mediated antiproliferative effect [35].

The role of NOS isoforms in NO production in response to proinflammatory cytokines has not been well defined. Our results show that iNOS, the expression of which is induced in our system after 24 h culture in the presence of proinflammatory cytokines, is implicated while constitutive isoform is not implicated in cytokine-induced antiproliferative response in *β* cells (Figure 4). Of note is that although iNOS inhibitor 1400 W inhibited the cytokine-mediated antiproliferative effect, its effect was only partial, and was unlike the total reversion by L-NMMA (a general NOS inhibitor). It is also interesting the effect of 1400 W increasing beta cell proliferation in absence of cytokines which could be due to a certain level of islet cells iNOS activation induced by islet isolation process. This partial effect of 1400 W suggests that other NOS isoforms are participating in antiproliferative response to cytokines. Endothelial NOS determination by western blotting was performed in cultured rat islets in the presence of proinflammatory cytokines and, contrary to the results reported by Lajoix et al. [2], eNOS expression was observed. This expression is maintained in culture and was not affected by the presence of cytokines (Figure 4(b)) and could be attributed to endothelial cells in the profuse vascular network present in pancreatic islets. Once the presence of eNOS in islets is confirmed, its implication in *β*-cell antiproliferative response to cytokines was tested in islet cultures treated with L-NIO [36] (an inhibitor whose primary target is eNOS) alone or in combination with cytokines. L-NIO induced a partial reversion of cytokine-induced *β*-cell antiproliferation in a similar manner to that induced by the iNOS inhibitor 1400 W. These results suggest participation of both NO isoforms in the antiproliferative response induced by proinflammatory cytokines, possibly by eNOS producing NO in a paracrine manner. In this sense, it has recently reported that inflammatory environment induces changes in eNOS which may adopt functional features of iNOS in a Ca^2+^-independent way [35].

To assess NO involvement in the pathophysiology of T1DM, we treated Biobreeding rats, an animal model of autoimmune diabetes, with the general NOS inhibitor L-NMMA at early stages of the insulitis process. Previous observations from our group indicated a halting of *β*-cell proliferation between 4 and 7 weeks of age and which can be reversed by administering neutralizing antibody against IFN-*γ* [37]. Treatment with L-NMMA of BB rats from 5 weeks of age induced a reversal of *β*-cell proliferation loss observed in untreated animals (Figure 5). In addition, a relative recovery of *β*-cell area with a regional pattern in treated animals at 7 weeks of age was observed. The effects, either on proliferation of *β* cell or on *β*-cell area, were very much more evident in the head than in the tail of the pancreas. A similar regional pattern of response was reported in earlier studies using a malnutrition model [38]. A plausible explanation for this regional effect could be a different vascularization in the head area compared to that in the tail which precluded equivalent drug availability in all areas of the pancreas volume. Further, a different response to environmental factors due to a different islet composition could underlie this observed effect [39]. This issue warrants further investigation.

In conclusion, T1DM prevention strategies focus, essentially, on early stages of the disease before clinical onset when there is enough *β*-cell mass in the pancreas to maintain its metabolic function. Our present study highlights the importance of NO as a mediator of the observed impaired *β*-cell proliferative response from the initial stages of insulitis. This impairment in proliferative capacity precludes an appropriate regenerative response of *β* cells to autoimmune damage. Our data also highlight the property of L-NMMA to maintain *β*-cell proliferative capacity in early insulitis stage and, as such, confirms that nitric oxide might be an important therapeutic target in the strategies of T1DM prevention.
